# Dentifrice

**Published:** 1876-03

**Authors:** 


### *DENTIFRICE.*

There is a much-advertised article for use upon the teeth, about which our opinion is often asked. Our answer has always been, "We have never used it, have never even seen it; so we are in no position to talk about it intelligently." It has, however, the confidence and commendation of many dentists, and members of this very useful profession are, by their training, the best judges of what is valuable in this direction.

We allude to the fluid called "Sozodont." We are told that it is a pleasant saponaceous liquid, aromatic and fragrant; and that it imparts a sense of purity and cleanliness to the mouth. If this is its character, nothing can properly be said against it. It were well if every person could be induced to use such an article as this, at least once each day. There would be no unclean and offensive mouths, no foul breath arising from accumulated food particles, and very few decayed teeth if such a dentifrice were universally used each night at bedtime.

Unquestionably the use of a solution of the choicest and most carefully compounded soaps upon the teeth has its specific advantage. The saliva of a healthy child, as well as that of all persons who live simply and wisely, is alkaline in its reaction. This alkaline quality tends to destroy the minute parasites which would otherwise live and thrive in contact with the teeth. But in many diseased conditions, in most diseasd conditions, in fact, the saliva loses its alkaline character and becomes acidulated. It is not uncommon to see a distinct groove, extending across two or more of the upper or lower teeth, and producing a very unpleasant deformity.

This groove simply indicates that at a certain period in childhood, when the impaired teeth had projected as far out of the gum as the grooved line, some infantile disease occurred, measels or scarlet-fever, or whooping-cough, or something which had acidulated the secretions. The acid saliva readily seized upon the lime of the imperfectly enameled teeth, and dissolved more or less of the structure. If at that time the mouth had been rinsed with a solution of soap, or any other mild alkaline fluid, the destructive action would have been arrested, and the natural contour and symmetry of the teeth preserved.

The alkaline fluids in the mouth of the perfectly natural liver, when in the vigor of youth and in robust health, is usually ample to save the teeth from those accumulations of infusoria which go to make up the offensive substance known as "tartar." But as civilization permits the existence of few if any of this entirely natural class, so few mouths are free from salivary calculus.

As the effect of this substance is to insinuate itself beneath the free edge of the gum, and to denude of its fleshy covering the portion of the teeth not protected by enamel ; to destroy the membrane which unites the tooth to the protecting tissues ; to induce ulceration and engorgement of the gum ; to cause absorption of the socket by ever-increasing pressure ; to impart a very unwholesome odor to every breath exhaled through the mouth, and to impregnate with a poisonous quality each breath inhaled through the same channels—it is important that active measures be resorted to to destroy these infinitesimal scourges as fast as they appear. To do this, a brush of good stiffness should be used, and should be charged with some mild alkali. A good dentifrice, having its alkaline ingredient nearly neutralized by the oily base employed, probably approaches as near to the perfect neutralizer of these conditions which permit and encourage infusorial life and its disgusting and injurious concomitants in the human mouth as can be found. This is said to be the quality which the dentifrice called " Sozodont " possesses.

